# What Is the Relationship Between Narcissism and Maladaptive Daydreaming? The Role of Defense Mechanisms as Mediators

**DOI:** 10.1176/appi.prcp.20250018

**Published:** 2025-04-09

**Authors:** Alessia Renzi, Rachele Mariani

**Affiliations:** ^1^ Department of Dynamic and Clinical Psychology and Health Studies “Sapienza” University of Rome Rome Italy

## Abstract

**Objective:**

Maladaptive daydreaming (MD) is a recently defined clinical condition characterized by an excessively immersive use of fantasy and imagination, which can significantly impact both professional and social life. The primary aim of this study is to examine potential associations between MD levels, narcissistic personality traits, and defense mechanisms. Additionally, the study aims to test mediation models to explore the direct and indirect roles of narcissism on MD levels through defense mechanisms.

**Methods:**

A total of 562 participants (mean age = 27.16 years; SD = 10.21; 68% female) completed an online survey comprising a socio‐demographic questionnaire, the Maladaptive Daydreaming Scale (MDS), the Pathological Narcissism Inventory (PNI), and the Defense Mechanisms Rating Scales‐Self Report‐30 item (DMRS‐SR‐30).

**Results:**

The MDS score was positively related to the PNI total and subscales. Moreover, there were negative associations with mature defenses, and a positive association with neurotic and immature defenses. Age showed a weak negative association with MDS. A direct effect of narcissism on MD levels and an indirect impact through immature and neurotic defense and direct trough mature defenses, emerged.

**Conclusions:**

The significant associations found in this study support a link between narcissistic personality traits and MD, where defenses seem to play a relevant role.

**Relevance to Clinical Practice:**

These findings underscore the potential benefits of treatments and clinical interventions aimed at fostering mature defense mechanisms. Such approaches can be particularly valuable for young adults in managing psychological distress linked to narcissistic vulnerability, which directly contributes to maladaptive fantasies and social withdrawal.

## Highlights

- Maladaptive daydreaming (MD) is a relatively recent condition, and research examining its connection to narcissism and defense mechanisms are needed.
- A positive association emerged between MD scores and both narcissism and neurotic and immature defenses, while a negative association was found with mature defenses.
- Defensive mechanisms emerged as mediators in the relationship between narcissism and MD.

Maladaptive daydreaming (MD) is a clinical condition characterized by an excessively immersive use of fantasy and imagination, involving detailed and vivid scenarios that significantly impact an individual's daily life (1, 2). Preliminary findings from a population study estimate that approximately 2.5% of people experience clinical‐level impairment due to these immersive daydreams (3). Additionally, MD shows a higher prevalence among young adults, peaking in the 18–30 age group (3). In MD, daydreaming may serve the function of providing access to experiences and emotions that may be missing in a person's real life, fostering empathy by engaging with imaginative scenarios (4, 5). Moreover, daydreaming can also serve as a protective mechanism for a fragile self‐image, offer comfort, allow for attentional redirection, provide an escape from distressing realities, or transform unfavorable situations within the realm of fantasy (6, 7, 8).

While daydreaming is often a normal emotional regulation strategy, some individuals may begin to rely on it in a rigid or addictive way, excessively or compulsively, thus consuming many hours each day immersed in their fantasy worlds, experiencing intense sensations of presence and vividness while maintaining a dual awareness that keeps them connected to the external world (2, 9, 10). Being deeply absorbed in detailed fantasies turns maladaptive when impairing academic, occupational, and social functioning (7, 10, 11).

MD syndrome is reported by people suffering from several disorders including attention deficit hyperactivity disorder, anxiety, depression, personality disorders, psychotic and disorders as well as different forms of addictions (7, 12). Some Authors suggest that MD is a specific and separate syndrome, distinct from other mental health conditions, yet sharing a similar functioning with other disorders such as dissociation, attention deficits, obsessive‐compulsive spectrum disorders, and behavioral addictions, with which MD frequently is in comorbidity (12, 13).

In this direction, accordingly to DSM‐5 a specific diagnostic criterion of Narcissistic Personality Disorder (NPD) includes considerations on the use of fantasy: “excessive involvement in fantasy about unlimited success, power, brilliance, beauty, or ideal love is a feature.” NPD is a personality trait characterized by an inflated self‐concept and behaviors aimed at preserving this self‐image despite reality (14, 15). Narcissistic individuals have deep‐seated feelings of inferiority along specific dimensions of the self, sense of entitlement and grandiose self‐relevant fantasies, but different interpersonal styles, that is, grandiose or vulnerable (7). Specifically, grandiose narcissism is associated with traits like grandiosity, aggression, and dominance, while vulnerable narcissism (VN) is defined by hypersensitivity to others' opinions, a strong need for approval, and defensiveness (14). Grandiose fantasies as well as the need for admiration represent common feature to both forms. Therefore, grandiose narcissists may use fantasy to maintain a grandiose self, whereas vulnerable narcissists, on the other hand, may avoid challenges and withdraw from situations which might reveal their real or imagined incompetence, weakness as well as alleviate their shame feelings through maladaptive self‐regulation including fantasy and grandiose daydreaming might help in managing a threatened sense of positive self‐evaluation (7, 14).

Only few studies explored the associations between MD and narcissistic personality finding significant correlations. More specifically Pietkiewicz et al. (7) found that more than 66% of people with NPD could be classified as maladaptive daydreamers, revealing also a strong relationship between MD and the VN. Similar findings emerged by Ghinassi et al.'s investigation (14) reporting that VN predicted MD through characterological shame. Grandiose narcissism was associated with MD to a lesser extent, and MD seems to be a dysfunctional but effective strategy to reduce characterological shame for grandiose narcissists—whilst the opposite seems to be true for vulnerable narcissists. In Morf and Rhodewalt (15), MDers reporting narcissistic grandiosity features used daydreaming as a means of wish‐fulfillment by fantasizing power and dominance, perpetrating physical and sexual violence, and capturing or rescuing someone. Although this is a relatively new area of investigation, previous findings suggest a significant relationship between MD and narcissistic personality traits, revealing that MD may be used as a potential defense against unpleasant emotional states. In this context, defense mechanisms are unconscious psychological processes that protect individuals from emotional pain and shield them from recognizing internal or external threats and stress (16). These mechanisms vary in maturity, leading to different levels of distortion in perceived reality (17, 18). According to a hierarchical model, defenses can be organized into three defensive categories going from a lower to a higher adaptiveness that include immature, neurotic and mature defenses respectively (19, 20). The immature defense category is the most extensive, encompassing all defenses related to action, denial, and image distortion operating by suppressing awareness of unacceptable thoughts, emotions, and behaviors, thereby protecting the individual from feelings of threat (19, 21). Relying heavily on immature defenses indicates a limited awareness of both the cognitive and emotional aspects of internal and external conflicts. The neurotic defense category includes defenses associated with neurotic and obsessional levels functioning keeping out of conscious awareness those parts of a conflict that would otherwise cause significant anxiety (19, 21). Individuals who frequently use these defenses can manage either the emotional or cognitive aspects of internal or external stressors, but typically handle only one aspect at a time. The mature defense category represents the most adaptive level, assisting individuals in managing psychologically stressful situations by integrating emotions with thoughts, thereby optimizing and potentially resolving the underlying internal or external sources of distress (19). Individuals typically employ a range of defense mechanisms to manage painful emotions, nevertheless the excessive reliance on immature and neurotic defenses can lead to negative outcomes such as psychological difficulties and psychopathology (22, 23, 24, 25). As regards the associations between MD and defense mechanisms, previous research has highlight in a robust and consistent manner the role of dissociation (2, 9, 26, 27, 28) since MD is marked by dissociative absorption, where attention is involuntarily focused to the exclusion of other internal and external factors (2, 9, 29). Accordingly, it has been suggested that MD may function as a way to distance oneself from a harsh and painful reality (25), including discomfort and inner fragility connected to pathological narcissism. However, in the international literature there is a paucity of study exploring the associations between MD and defense mechanisms in general. In this direction, Musetti et al. (23) highlighted positive associations between MD scores and immature and neurotic defense as well as no significances for mature defenses.

## The Present Study

Given this background MD is a quite recent conditions and studies connecting this syndrome with pathological narcissism and defense mechanism are still limited. The general aim of present study is to contribute to deepen the knowledge on MD, precisely the first aim is to explore the associations between MD, narcissistic personality traits, defense mechanisms, age and gender. A further aim is to test the role of immature, neurotic and mature defenses in mediating the associations between narcissism and MD. We hypothesize significant positive correlations between MD and both narcissistic personality traits as well as immature and neurotic defenses, as a greater reliance on these types of defense mechanisms may serve as a risk factor for increased use of maladaptive fantasies. Conversely, we expect a negative association between MD and mature defenses, as mature defense mechanisms could offer protection against persistent and dysfunctional absorption in daydreaming. Regarding the mediation models, we hypothesize an indirect effect of narcissism on MD through defense mechanisms.

## METHODS

### Participant and Procedure

This study was conducted from November 2023 to March 2024 and adhered to the ethical guidelines of the World Medical Association (Declaration of Helsinki) for research involving human subjects. Ethical approval was obtained from our University Department's Ethics Committee. To ensure consistent participation, an online survey was created using the Typeform platform in English. The survey was distributed across various social media platforms (Facebook, Instagram, etc.) and websites popular among young adults, as MD is common in this demographic. Participants provided informed consent before completing the survey. The inclusion criteria were: (a) Age between 18 and 60 years; (b) Fluency in English; (c) Provision of informed consent.

A total of 605 individuals completed the survey, but 43 responses (7.1%) were excluded due to duplication, inconsistent answers (e.g., the same response for all questions), or failure to meet the inclusion criteria (e.g., age outside the specified range). The final sample consisted of 562 participants, with a mean age of 27.16 years (SD = 10.21), of whom 68% were female. Regarding the nationality of participants, the majority were Italian (35.5%), followed by Greek (18%), Turkish (7.5%), and Spanish (7%). 32% of the participants fall into the “others” category, which encompasses various nationalities that, individually, do not have significant representation. Table 1 provides an overview of the group's sociodemographic characteristics as well as the mean levels of the psychological dimensions investigated (see Table 1).

**TABLE 1:** Participants' sociodemographic characteristics.^a^

Variables	*N*	(%)
Gender		
Female	382	68.0
Male	180	32.0
Nationality		
Italian	200	35.7
Greek	100	18
Turkish	42	7.5
Spanish	37	7
Other	183	32
Civil status		
Married	59	10.6
Single	412	73.2
Divorced	16	2.8
Cohabitant	75	13.4
Children		
No	500	88.7
Yes	62	11.3
Educational level		
High school	165	29.5
Student college	115	20.5
Bachelors	180	32.1
Master	86	15.2
PhD and above	16	2.8
Employment status		
Student	305	54.3
Employed	191	34.0
Freelance	36	6.3
Not occupied	30	5.3
Drugs assumption		
No	457	81.1
Yes	105	18.9
Current psychotherapy		
No	449	79.7
Yes	113	20.3

	Mean	SD
MDS‐16	29.91	18.52
PNI‐total	2.47	0.73
Mature defense	37.30	9.44
Neurotic defense	23.84	4.92
Immature defense	38.84	8.06
Immature non‐depressive defense	16.06	4.72
Immature depressive defense	22.78	6.86

^a^ MDS‐16, Maladaptive Daydreaming Scale‐16 item; PNI, Pathological Narcissism Inventory.

### Measures

#### Socio‐Demographic Questionnaire

It was used to gather data on demographic variables, including age, gender, educational level, marital and employment status, nationality, presence of children, engagement in psychotherapy, and medication use.

#### The 16‐Item MDS

The 16‐item Maladaptive Daydreaming Scale (MDS) (1, 2) is a self‐report instrument with 16 items designed to assess MD. Participants rate their experiences on a scale from 0 (never/none of the time) to 100% (extremely frequent/all the time), in 10% intervals. The questionnaire's creators recommend a cut‐off score of 51 to differentiate between those with and without self‐diagnosed MD. The instrument has demonstrated strong psychometric properties (1, 2). In the current study, the overall scale had a Cronbach's alpha of 0.92.

#### Defense Mechanism Rating Scales‐Self‐Report

The Defense Mechanism Rating Scales‐Self‐Report (DMRS‐SR‐30) (30): This instrument consists of 30 items, each rated on a 5‐point Likert scale from 0 (not at all) to 4 (very often/much). It assesses 30 individual defense mechanisms categorized into mature, neurotic, and immature defenses, with the latter further divided into depressive and non‐depressive immature defenses. Higher scores indicate a greater reliance on that type of defense. In the current study, the Cronbach's alpha values for this measure were 0.86 for neurotic defenses and 0.92 for immature defenses. [Correction added on 26 April 2025, after first online publication: In this paragraph, reference citation ‘19’ was made in error and has been changed to ‘30’, which has been added to the reference list. All subsequent references and citations were renumbered.]

#### Pathological Narcissism Inventory

The Pathological Narcissism Inventory (PNI) (31) it is a self‐report measure consisting of 52 items, rated on a 6‐point Likert scale from 0 (not at all like me) to 5 (much like me). This instrument explores 7 dimensions of pathological narcissism: (1) Entitlement Rage, (2) Exploitativeness, (3) Grandiose Fantasy, (4) Self‐sacrificing Self‐enhancement (all associated with narcissistic grandiosity), (5) Contingent Self‐Esteem, (6) Hiding the Self, and (7) Devaluing (all associated with narcissistic vulnerability). Higher scores indicate a greater level of pathological narcissism. In the current study, the Cronbach's alpha for the total score was 0.94.

### Statistical Analysis

All statistical analyses were conducted using the Statistical Package for the Social Sciences (SPSS) version 25 for Windows (IBM). Data were presented as frequencies and percentages for categorical variables, and as means and standard deviations for continuous variables. Pearson's correlation analysis was used to assess the relationships between psychological variables, as well as age and gender. Mediation analyses were carried out to investigate both the direct effect of narcissism on MD levels and the indirect effect through neurotic, immature and mature defenses, respectively. Age was included as a covariate in the models. Mediation models were tested using the SPSS macro, PROCESS, with linear regression models fitted and the size and significance of the indirect effects estimated using a bootstrap procedure. A *p*‐value of <0.05 was considered statistically significant.

## RESULTS

29% of the participants obtained a total score of 40 or higher, exceeding the clinical cut‐off for MDS. Correlation analysis revealed significant associations between the MDS and all the psychological dimensions investigated (see Table 2). Specifically, there were positive associations between MDS with the total score and subscales of the PNI, as well as with neurotic and immature defenses, included non‐depressive and depressive ones. Conversely, negative association was observed with mature defenses. A weak negative association between MDS with age was found, while no significant associations with gender were observed.

**TABLE 2:** Correlations between MDS‐16 scores and narcissisms, defense mechanisms and demographic variable.^a^

	MDS‐16
Age	−0.183*
Gender	−0.004
PNI‐total	0.401**
PNI‐entitlement rage	0.272**
PNI‐exploitativeness	0.216**
PNI‐grandiose fantasy	0.353**
PNI‐self‐sacrificing self‐enhancement	0.260**
PNI‐contingent self‐esteem	0.300**
PNI‐hiding the self	0.249**
PNI‐devaluing	0.335**
DMRS‐SR‐30	
Mature defense	−0.302**
Neurotic defense	0.127**
Immature defense	0.276**
Immature non‐depressive defense	0.233**
Immature depressive defense	0.164**

^a^ DMRS‐SR‐30, Defense Mechanism Rating Scale‐Self report‐30 item; MDS‐16, Maladaptive Daydreaming Scale‐16 item; PNI, Pathological Narcissism Inventory.

*p < 0.05, **p < 0.01.

**FIGURE 1:**
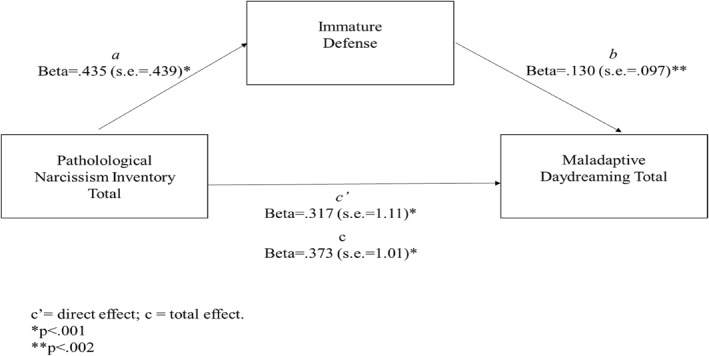
Mediation model using immature defense as mediator.

Model 4 of the PROCESS macro (32) was used to investigate whether the indirect effect of immature defense on the link between pathological narcissism (PNI total) and MD (MDS) was significant (see Figure 1). Age was considered a covariate. Results showed a significant total effect of narcissism symptomatology on MDS levels (*b* = 9.420; SE = 1.010; *β* = 0.373; *p* < 0.0001). Moreover, results showed a significant direct effect of narcissistic symptomatology (PNI total) on immature defense (*b* = 4.812; SE = 0.439; *β* = 0.4355; *p* < 0.0001) but not of age (*b* = 0.0178; SE = 0.031; *β* = 0.023; *p* = 0.568). The direct effect of the immature defense on the MDS scores was also significant (*b* = 0.296; SE = 0.097; *β* = 0.130; *p* = 0.002) as well as the direct effect of the narcissistic symptomatology on the MDS level (*b* = 7.999; SE = 1.110; *β* = 0.317; *p* < 0.0001), with a significant value emerging for age (*b* = −0.162, SE = 0.071, *β* = −0.091; *p* = 0.02). Finally, the indirect effect was significant (*b* = 1.428, *β* = 056; bootstrap SE = 0.186, bootstrap 95% C.I.: 0.0216–0.0941). Therefore, narcissistic traits demonstrated also an indirect effect on MD levels through immature defense. The overall model accounted for about 18% of the variance in MDS scores, *R* ^2^ = 0.178, *F*_(3, 553)_ = 40.032; *p* < 0.0001.

**FIGURE 2:**
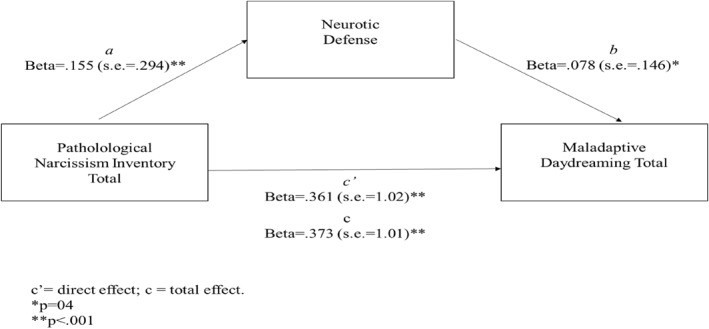
Mediation model using neurotic defense as mediator.

A further model was tested to investigate whether the indirect effect of neurotic defense on the link between narcissistic personality traits (PNI total) and MD (MDS) was significant (see Figure 2). Age was considered a covariate. Results showed a significant total effect of narcissistic symptomatology on MDS levels (*b* = 9.420; SE = 1.014; *β* = 0.373; *p* < 0.0001). Moreover, results showed a significant direct effect of narcissistic symptomatology (PNI total) on neurotic defense (*b* = 1.053; SE = 0.294; *β* = 0.1557; *p* < 0.001) but not of age (*b* = 0.0125; SE = 0.020; *β* = 0.053; *p* = 0.217). The direct effect of the neurotic defense on the MDS scores was also significant (*b* = 0.292; SE = 0.146; *β* = 0.078; *p* = 0.04) as well as the direct effect of the narcissistic symptomatology on the MDS level (*b* = 9.112; SE = 1.022; *β* = 0.361; *p* < 0.0001), with a significant value emerging for age (*b* = −0.164, SE = 0.071, *β* = −0.092; *p* = 0.02). Finally, the indirect effect was significant (*b* = 0.307, *β* = 012; bootstrap SE = 0.006, bootstrap 95% C.I.: 0.0006–0.0272). Therefore, narcissism demonstrated also an indirect effect on MD levels through neurotic defense. The overall model accounted for about 17% of the variance in MDS scores, *R* ^2^ = 0.17, *F*(3, 553) = 37.9226; *p* < 0.0001.

**FIGURE 3:**
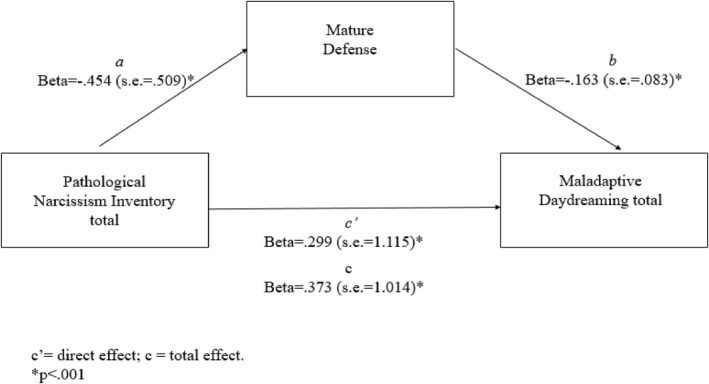
Mediation model using mature defense as mediator.

A last model was tested to investigate whether the indirect effect of mature defense on the association between narcissism (PNI total) and MD (MDS) was significant (see Figure 3). Age was considered a covariate. Results showed a significant total effect of narcissistic symptomatology on MDS levels (*b* = 9.420; SE = 1.014; *β* = 0.373; *p* < 0.0001). Moreover, results showed a significant direct effect of narcissistic symptomatology (PNI total) on mature defense (*b* = −5.865; SE = 0.509; *β* = −0.454; *p* < 0.001) but not of age (*b* = 0.0125; SE = 0.020; *β* = 0.053; *p* = 0.217). The direct effect of the mature defense on the MDS scores was also significant (*b* = −0.318; SE = 0.083; *β* = −0.163; *p* = 0.001) as well as the direct effect of the narcissistic symptomatology on the MDS level (*b* = 7.553; SE = 1.115; *β* = 0.299; *p* < 0.0001), with a significant value emerging for age (*b* = −0.172, SE = 0.071, *β* = −0.095; *p* = 0.02). Finally, the indirect effect was significant (*b* = 1.867, *β* = 074; bootstrap SE = 0.018, bootstrap 95% C.I.: 0.0389–0.1109). Therefore, narcissism demonstrated also an indirect effect on MD levels through neurotic defense. The overall model accounted for about 19% of the variance in MDS scores, *R* ^2^ = 0.186, *F*(3, 553) = 42.110; *p* < 0.0001.

## DISCUSSION

In our study, we explored the relationship between MD and narcissism, attempting to investigate the role of defense mechanisms as mediators of the relationship.

The results of the correlations between the narcissism and MDSs show a close and linear relationship between the two constructs with all subscales being significant, not just one subtype of symptoms. Narcissism therefore both in its grandiose aspects and in its more intimate manifestations presents a close relationship with the controlled creation of daytime fantasies. This result is broadly consistent with previous studies supporting an association between MD and narcissistic personality (7, 14, 33). However, a predominant role of vulnerable characteristics over grandiose characteristics in relation to MD did not emerge with PNI scales contributing in determining this two narcissistic dimensions showing similar correlational values, and this partially contrasts with earlier research (7, 14, 33). The relationship between maladaptive fantasies and narcissistic psychopathology has always been supported by psychodynamic theories that have emphasized the powerful role played by the creation of substitute fantasies to reality as a process of self‐regulation (34, 35) specially sexual fantasies in adolescence (36). Our data confirm this relationship especially MD may be the manifestation of an attempt to compensate for a deep sense of inferiority and ineffectiveness in young adults. Not only traumatic and dissociative aspects can create this need to regulate these internal aspects but also an inadequate sense of self to cope with reality.

NPD is conceptualized by Kernberg as one of the levels of personality, characterized by an unstable identity and primitive defense mechanisms in the stabilization of object relations. In our study we explore the role of defense mechanism related to NPD and MD (37). Correlations between defense mechanisms and MD show an interesting relationship between mature defenses as protective functioning of the MD symptom. In fact, there is a significant inverse correlation between mature defenses and MD, and a significant linear correlation with immature and neurotic defenses. Interestingly neurotic defuses resulted to have only a weak and marginal association with MD, thus a most relevant association, and presumably predictive role, seems to be played by immature and mature ones. The protective aspect of mature defenses appears to be an innovative element in our work compared to a previous studies (23).

As regards the association between MD and the anamnestic variables investigated, the results obtained show a weak but negative relationship between MD and age of the participants, but no difference by gender. The literature highlights age as a specific factor, indicating that daydreaming is more prevalent among younger individuals. Therefore, present finding is in line with the broader literature highlighting how young age can be a risk factor for MD condition (3, 28). On the other hand, in our investigation there is no evidence of a gender difference and this in line with a recent previous studies failing in finding a clear pattern that would suggest higher MD prevalence among females (3).

Through three mediation models, we aimed at exploring the role of immature, neurotic and mature defenses respectively, in mediating the associations between narcissistic personality traits and MD. All these models resulted significant with narcissism showing both a direct and indirect effect through the defenses tested. In Gholami Zarch et al. (38) the mediating role of mature defense did not emerged whereas in present study a protective role emerged as well as a risk role for the immature and neurotic. Our results are in line with a recent study (38) that highlighted how mature defenses are direct mediators of the construction of integrated object relations by reducing narcissistic traits. These findings highlight the close link between maladaptive diurnal fantasies, narcissistic fragilities and the role of internal mechanisms in regulating one's adaptive processes to reality.

### Limitations

While this study provides several new insights into MD, there are several limitations to acknowledge: (a) The use of a “snowball” sampling method through institutional websites and social media may introduce selection bias; (b) Dependence on self‐report questionnaires could lead to response bias if participants inaccurately represent their experiences; (c) The survey was administered in English, which may not be the first language for all participants; (d) The use of instruments evaluating respectively the narcissism and the defense mechanism that are based on a dimensional approach not providing information about individuals' most representative category of defense mechanism as well as no information regarding pathological/not pathological narcissism personality. Moreover, regarding narcissism, the use of an instrument that specifically provides distinct measures of grandiose and VN seems desirable, as it may help to further explore the association between these two dimensions and MD, as well as their relationship with defensive functioning. Therefore, future studies employing categorical measures may be beneficial; (e) The cross‐sectional nature of the study means that findings should be interpreted with caution; (f) The lack of inclusion of other relevant psychological constructs, such as affect regulation capabilities, which may play a significant role in MD syndrome. Future studies that also consider this dimension would be important. Although the mediation analyses offer a deeper understanding of the relationships between variables, they do not establish causation. Future longitudinal research is necessary to validate and expand upon these preliminary findings.

## CONCLUSIONS

Although the limitations mentioned above exist, the present study also has strengths, including its exploration of a relatively novel area of investigation, its related findings, and the substantial sample size obtained. Notably, our results confirm a significant and meaningful relationship between narcissistic personality and MD. Above all, our results highlight an important mediating role played by the individual's defensive functioning. Neurotic and immature defenses explain part of the relationship between narcissism and MD by highlighting their direct relationship, while mature defenses are inversely related to narcissism and MD by playing a protective role in the individual's psychic functioning. This relevant result highlights how possible treatments and clinical interventions that promote the use of mature defense mechanisms can help people, especially young adults, to cope with psychological distress caused by narcissistic fragility, which has a direct effect on phantasmatic and maladaptive withdrawal. In this light, treatments with a psychodynamic orientation (such as defense‐based therapies, transference‐focus therapy or mentalizing‐based therapy) seem best suited to facilitate the dimension of reflecting on one's defensive dynamics and promoting the use of mature ones.
